# Short and long-term effectiveness of couple counselling: a study protocol

**DOI:** 10.1186/1471-2458-12-735

**Published:** 2012-09-03

**Authors:** Margot J Schofield, Nicholas Mumford, Dubravko Jurkovic, Ivancica Jurkovic, Andrew Bickerdike

**Affiliations:** 1 School of Public Health, La Trobe University, Melbourne, VIC 3086, Australia; 2 Relationships Australia Victoria, 450 Burke Road, Camberwell, VIC, 3124, Australia

**Keywords:** Couple counselling, Relationship education, Marital satisfaction, Relationship commitment, Depression, Effectiveness, Health outcomes, Community services

## Abstract

**Background:**

Healthy couple relationships are fundamental to a healthy society, whereas relationship breakdown and discord are linked to a wide range of negative health and wellbeing outcomes. Two types of relationship services (couple counselling and relationship education) have demonstrated efficacy in many controlled studies but evidence of the effectiveness of community-based relationship services has lagged behind. This study protocol describes an effectiveness evaluation of the two types of community-based relationship services. The aims of the Evaluation of Couple Counselling study are to: map the profiles of clients seeking agency-based couple counselling and relationship enhancement programs in terms of socio-demographic, relationship, health, and health service use indicators; to determine 3 and 12-month outcomes for relationship satisfaction, commitment, and depression; and determine relative contributions of client and therapy factors to outcomes.

**Methods/Design:**

A quasi-experimental pre-post-post evaluation design is used to assess outcomes for couples presenting for the two types of community-based relationship services. The longitudinal design involves a pre-treatment survey and two follow-up surveys at 3- and 12-months post-intervention. The study is set in eight Relationships Australia Victoria centres, across metropolitan, outer suburbs, and regional/rural sites. Relationships Australia, a non-government organisation, is the largest provider of couple counselling and relationship services in Australia. The key outcomes are couple satisfaction, relationship commitment, and depression measured by the CESD-10. Multi-level modelling will be used to account for the dyadic nature of couple data.

**Discussion:**

The study protocol describes the first large scale investigation of the effectiveness of two types of relationship services to be conducted in Australia. Its significance lies in providing more detailed profiles of couples who seek relationship services, in evaluating both 3 and 12-month relationship and health outcomes, and in determining factors that best predict improvements. It builds on prior research by using a naturalistic sample, an effectiveness research design, a more robust measure of relationship satisfaction, robust health indicators, a 12-month follow-up period, and a more rigorous statistical procedure suitable for dyadic data. Findings will provide a more precise description of those seeking relationship services and factors associated with improved relationship and health outcomes.

## Background

The couple relationship forms a fundamental stabilising unit in society, with 64% of Australian adults living in couple relationships in 2010, 53% in registered marriage and 11% in de facto relationships [1]. Nevertheless, there is an increasing divorce rate of more than 50% in most developed countries [2]. In Australia, the median length of marriage before separation is 8.8 years, and approximately half of all divorces involve couples with children [1].

These high rates of relationship breakdown have been consistently associated with negative health consequences for both adults and children following divorce/separation. These include isolation from support networks, and reduced income and standard of living for both adults and children [3], dilemmas of loyalty over children for men, and depression and loss of identity for women [4,5]. In a meta-analysis of 70 US studies, children of divorce scored significantly lower on measures of academic achievement, conduct, psychological adjustment, and social development [6]. Longitudinal studies also suggest that children of divorce have a higher incidence of psychological disorders, drug and alcohol use, and risky sexual behaviour [7].

Although the effects of divorce and separation can be detrimental, research indicates that high relationship discord in intact couples is also likely to have negative outcomes. For example, a large-scale study (n = 2,677) in the US found that relationship discord, regardless of marital status, significantly predicted a higher incidence of mental disorders such as mood and anxiety disorders in adults and negative social outcomes [8]. Specifically, high discord was associated with lower social interactivity with family and friends, and lower work satisfaction [8]. These results are congruent with those from previous reviews [9,10]. Therefore, merely ‘staying together’ is unlikely to prevent negative outcomes if relationship discord and conflict persist. Such findings indicate a pressing need for research that evaluates relationship services designed to improve relationship quality. In this study we focus on couple counselling and relationship enhancement/education programs. Furthermore, factors that influence the outcomes of these services need thorough investigation. Research to date has identified both couple and individual factors that may contribute to relationship discord. These include relationship satisfaction and commitment at the couple level, and depression at the individual level. However, robust research to evaluate relationship-enhancing interventions in the community are scarce. This paper presents the study protocol for a naturalistic longitudinal study conducted in Victoria Australia, the Evaluation of Couple Counselling (ECC) study, and describes how it addresses current gaps in the research literature.

### Key relationship constructs

#### Relationship satisfaction

Relationship satisfaction has been the most common outcome variable identified in more than 200 evaluations of couple counselling [11,12]. Studies have found significant improvements in relationship satisfaction from pre- to post-treatment [13,14] and over the course of one to two years following counselling [15]. In these studies, relationship satisfaction was most frequently assessed using the Dyadic Adjustment Scale (DAS) [16]. However, some researchers suggest that the DAS is too broad in its scope, and specific measures of relationship satisfaction, such as the more recently developed Couple Satisfaction Index (CSI), should be used for precise assessment [17,18]. Therefore, while most studies indicate improvements in relationship satisfaction following couple counselling, they are limited by the samples and measures used, largely short-term follow-up time frames, and analyses that do not account for the dyadic nature of couple data.

#### Relationship commitment

Relationship commitment, based on measures such as the Commitment Inventory (CI) [19], is another commonly investigated relationship outcome. Commitment is conceptualised as a combination of partners’ ‘want’ and ‘need’ to stay in their relationship [19]. An individual’s ‘want’ to stay together represents how much they care for their partner, and desire for the relationship to continue. Conversely, the ‘need’ to stay in a relationship refers to practical reasons to avoid separation (e.g., to avoid financial burdens). Accordingly, outcome research has indicated a positive relationship between improving relationship commitment through couple counselling and improvements in relationship satisfaction [13,20-22]. Therefore, relationship commitment should be considered as a potentially influential factor in future evaluations of relationship services.

#### Depression

Importantly, relationship discord has been associated more recently with the occurrence of depression in at least one partner [23]. Theorists assert that a bi-directional association between depression and relationship discord may exist [24], based on research showing that reducing depression (assessed by standardised measures such as the Beck Depression Inventory, and the Center for Epidemiological Studies Depression Scale) can significantly predict the success of couple counselling (for reviews, see [25-27]). The consistency of these findings indicates that depression may play a significant role in determining outcomes of couple counselling, and warrants further study.

To summarise, research indicates that couple-specific variables as well as individual factors may predict the outcomes of couple counselling and relationship services. The causal direction of these relationships, however, is less clear. These observations are important, since, to justify and guide the application of relationship services such as couple counselling, empirical evidence must explore both the outcomes of relationship services and the factors that predict successful therapy.

### Evaluation of relationship services

Relationship services are offered in a complex psychosocial and service environment. Consistent with definitions of evidence based practice [28], multiple sources of evidence need to be processed for effective clinical decision making [29-31]. While efficacy studies using randomized controlled trial designs are highly valued in evidence-based practice environments [28], they are rarely feasible or ethical when couples are seeking relationship services, often in heightened states of distress or urgency [32]. Furthermore, efficacy findings do not necessarily translate to naturalistic, community-based settings [32]. Therefore, effectiveness studies, which are less controlled, are often more appropriate for evaluating outcomes in mental health agencies [30]. In the current couple counselling research literature, there is an imbalance in favour of efficacy studies, with little evidence available for effectiveness of services in community-based settings [33]. This study focuses on the evaluation of two types of services: couple counselling and relationship enhancement services

### Couple counselling outcome studies

In evaluating the outcomes of couple counselling, earlier efficacy studies have outlined several therapies that may be considered ‘efficacious’ treatments. For example, Behavioural Marital/Couple Therapy and Cognitive Behavioural Therapy-based couple counselling have significantly reduced relationship distress, as measured by the Marital Adjustment Scale (MAT) [34], and the DAS [35,36], with results maintained over time, and compared to no-treatment controls [37,38]. Emotion Focussed Therapy has demonstrated similar results, and is considered efficacious [26,38,39]. However, such efficacy studies have been criticised for lacking the external validity necessary for application in day-to-day clinical practice [32]. In particular, their adherence to manualised treatments is seen as a limitation, since this may not sufficiently represent therapeutic competence [40]. A further dilemma is that efficacy research evidence exists largely for counselling ‘difficult’ populations, such as those with a diagnosis of major depression, rather than more common client groups seen in typical counselling settings [41,42]. Therefore, there is a growing consensus that efficacy studies should be complemented by effectiveness research to best inform clinical practice [29].

The limited effectiveness research that exists to date suggests that couple counselling can improve outcomes such as relationship satisfaction [33,43], communication skills and general well-being [44], at least in some European countries. No community-based effectiveness research has been undertaken in Australia. Accordingly, there is a pressing need for effectiveness research examining the outcomes of couple counselling in different community-based settings. Increasing the number, and broadening the type of settings, of these studies will provide more robust evidence of the effectiveness of community-based couple counselling. If found to be effective across a range of settings and cultural contexts, the data will support advocacy for better funding for couple counselling, and inform future studies that seek to define the effective ingredients.

### Relationship education outcome studies

Couples wanting to improve their relationship may access other forms of relationship services, the most common of these being relationship education programs. We currently know little about the profiles of couples who seek out relationship education compared with those who seek relationship counselling, or the outcomes of these programs. However, anecdotal evidence suggests that there may be considerable distress among at least some couples seeking relationship education.

Relationship education programs differ from couple counselling as they are typically highly structured, conducted in groups, and focus on a mixture of four components; awareness, feedback, cognitive change, and skills training [45]. Firstly, ‘awareness’ helps couples clarify their expectations in the relationship. Feedback involves participants completing questionnaires about their relationship (e.g. measures of interpersonal problems), and receiving information on what their scores indicate. Cognitive-behavioural approaches promote changing cognitions to facilitate positive relationships. These may include promoting realistic attributions/expectations around negative partner behaviour [46]. Finally, in skills training, couples attend lectures or presentations on relationship skills, and practise these during facilitator-led activities [45].

Two recent meta-analyses of 97 and 114 outcome studies [47] have found moderate effect sizes for improving relationship quality, and couple’s communication following relationship education programs. These effects have persisted for up to 4 years in some studies [47]. However, these meta-analyses highlight limitations in the current literature on relationship education. Specifically, the majority of studies involved couples from upper socio-economic backgrounds who were not experiencing high relationship discord [47,48]. This sample profile may not represent clients who typically present for relationship education. Thus, further investigation of relationship education services is required to inform subsequent research, and service delivery.

Very little research has examined the comparative benefits of couple counselling and relationship education programs. As clients are likely to self-select into these service types, it is not clear whether characteristic relationship distress profiles present to each service type, or indeed whether there is an interaction between presenting profile, service type and outcome.

#### Aims of the current study

In the Effectiveness of Couple Counselling (ECC) study, we aim to conduct an evaluation of couple counselling and relationship education services. We propose first to map and compare the relationship, health and wellbeing profiles of couples attending couple counselling and relationship education services in community-based settings in Australia, and to examine the factors associated with better relationship satisfaction and general wellbeing in both groups. Second, we aim to assess the outcomes of both couple counselling and relationship enhancement services over both short- and long-term (3 and 12 months), and to clarify the characteristics which best determine improved couple and individual outcomes in both groups. Previous outcome studies have shown that clients, on average, continue to improve significantly after treatment, providing a strong case for longer-term follow-up [42,49]. Thus, we have included a 12-month follow-up to gauge longer-term trends and effects.

The study uses a number of standardized outcome measures since some prior investigations have been criticised for their lack of standardised assessment [50]. Finally, the use of statistical analyses that assume independence of data, such as *t*-tests, or ANOVAs, has been prevalent in previous studies [44,49]. Unfortunately, this assumption is rarely tenable for couple data [51]. Therefore, we propose to utilise multi-level statistical modelling procedures that control for the inter-dependence of couple data to assess any treatment effects.

The specific aims of the ECC study are to:

1. Map profiles of clients seeking community agency-based couple counselling vs. relationship enhancement programs in terms of socio-demographic and relationship indicators (such as relationship satisfaction, relationship commitment, interpersonal problems, and reasons for attending), as well as health (such as depression, general wellbeing) and health service use (eg. use of medical services) factors.

2. Determine whether couple counselling and relationship education services improve three- and twelve-month outcomes for relationship satisfaction, commitment, and depression, using statistical analyses appropriate to couple data.

3. Determine the relative contributions of client factors (individual and couple) and therapy/education factors to outcomes at 3- and 12-months, and to sustainability of outcomes over time.

Table 1 provides further details on the aims of the ECC study and the key features of the study protocol in relation to these aims.

**Table 1:** Research aims, methods of addressing these, and potential benefits

**Research aim**	**Methods**	**Benefit**
1. To map profiles of clients seeking agency-based couple counselling vs. relationship enhancement programs in terms of relationship indicators, as well as socio-demographic factors, health and health service use.	Statistical comparison of pre-counselling data from couple counselling vs relationship education groups. Multi-level modelling to describe relationships between variables controlling for dyadic (couple) data.	To increase knowledge of ‘who’ attends counselling and relationship education programs, and presenting issues. This will guide development of clinical and educational approaches, and professional training. Comparative analyses will determine whether couples attending the two services differ on key variables. Such knowledge can better inform clinicians about couples’ needs.
2. To determine whether couple counselling improves 3 and 12-month outcomes for relationship satisfaction, commitment, and depression, using statistical analyses appropriate to couple data.	Multi-level modelling to determine pre-post differences, controlling for dyadic (couple) level.	To contribute to the literature assessing the effectiveness of community-based couple counselling. The results will assist clinical decision-making in community-based relationship service settings, and professional training.
3. To determine the relative contributions of client/couple and therapy factors to outcomes at 3- and 12-months, and to sustainability of outcomes over time.	Multilevel statistical modelling of key predictors of relationship and individual outcomes controlling for dyadic data.	To increase knowledge of factors influencing relationship services outcomes. This information will inform the training and development of clinicians and educators, and tailoring of relationship treatments to couples.

## Methods/Design

### Design

The ECC study is designed as a quasi-experimental pre-post-post evaluation of couples presenting for two types of community-based relationship services: couple counselling and relationship education groups. As appropriate for an effectiveness design, couple counselling was provided as usual by the agency counsellors. The relationship education course involved the standard eight-session relationship skills workshops called Good Connecting (GC) offered by the same community-based agencies. Using this naturalistic design, participants ‘self-select’ into either group as random allocation is not feasible in this setting. This naturalistic design is appropriate for descriptive and effectiveness studies [30], and will allow us to build evidence based on current practice.

While inclusion of a no-treatment comparison group is considered an important characteristic of a well-controlled evaluation design, ethical issues preclude withholding treatment from distressed couples in effectiveness studies. Indeed, some researchers now argue that including a no treatment control group for couple counselling research is redundant [16,52]. Baucom and colleagues (2003) reviewed 17 studies that assessed couple counselling using a control group, and found no change in relationship quality for the control groups (overall effect sizes between 0 and 0.1). These authors conclude that future research may use a single treatment group under the assumption that no-treatment control groups do not demonstrate improvement.

**Figure 1:**
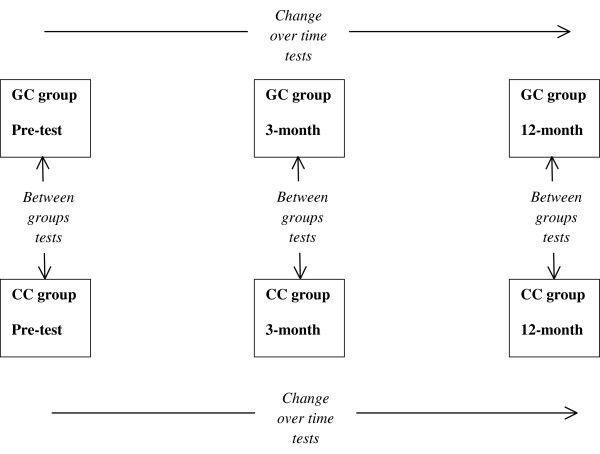
Design of the EEC study, and planned data analyses.

Our longitudinal design, shown in Figure 1, involves a pre-treatment survey following recruitment in 2008–2009, and follow-up surveys at 3- and 12-months post-intervention from 2009–2011. All consenting couples beginning an intervention will be included in the study, regardless of number of sessions completed.

### Setting

The setting for this study is the network of eight locations of Relationships Australia Victoria (RAV). Three sites are in the Melbourne metropolitan area, two in Melbourne outer suburbs, and three in regional and rural centres of Victoria. Relationships Australia, a secular non-government organisation, is the largest provider of couple counselling and relationship services in Australia and has been providing relationships services to the Victorian community for over 65 years Their services are offered to a diverse range of clients, with government subsidies for low income couples. Therefore, the setting for our effectiveness study has high ecological validity. This strengthens the applicability of our findings as they come from well-established, ‘working’ relationship counselling agencies, which are partially supported by considerable government funding.

### Sample

Our cohort samples comprise participants presenting for either couple counselling or the GC course at the eight participating RAV agencies. The counselling group sampling criteria are that couples must express willingness to attend the first session together, be seeking counselling to improve their relationship, and be English-speaking. After the first session, the counsellors or educators further assess eligibility and recommend excluding the following: those intending to dissolve their relationship, couples where violence is currently occurring, and those involving a serious mental health issue requiring individual assessment and treatment. Our sampling procedure aimed to recruit both partners in each couple, but accepted one member of a couple if their partner attended the service but did not consent to take part in the research. All couples presenting for relationship enhancement were considered eligible.

#### Power analysis

For the long-term evaluation, a power analysis was conducted as follows. The eight RAV sites provide counselling services to approximately 2,000 new couples per year, with an estimated 70% (1,400 couples) being eligible for the study. Based on a conservative consent rate of 20%, an approach to all presenting couples over a year would be necessary to gain a consenting sample of 280 couples. An estimated 10% were likely to be declared ineligible following the initial session, yielding approximately 252 eligible couples at baseline. Based on historical RAV data, we anticipated a 20% attrition rate by 3 month follow-up (n = 201 couples) and a further 30% by 12 month follow-up, yielding a final evaluation sample of 141 couples. This is sufficient to predict key outcomes using 10 predictor variables, with 80% power and a .05 significance level. Similar numbers were anticipated for the relationship education sample.

### Recruitment and procedures

Strong support from the central management at RAV was obtained from the outset, and their research director (AB) was involved in the design of the study and promoting staff cooperation. In attempting to meet our recruitment goals, a three-stage recruitment and data collection process was employed.

#### Stage 1: Introduction of research to agency staff

The first stage involved introducing the research to staff in each of the eight agencies, and discussing potential benefits of the study. The aims were to encourage the cooperation of administrative and service delivery staff as integral in the recruitment process, and promote the relevance of the research for their day-to-day practice. Through negotiation, recruitment processes were integrated as closely as possible into usual practice within each agency.

#### Stage 2: Recruitment of participants

When eligible clients first contacted the agency to make an appointment, the reception staff informed them about the research, and gained consent to send out an information package in the mail. This included an introduction letter, a description of the study, consent forms, pre-counselling questionnaires, two reply-paid envelopes for separate return of the forms and questionnaires by each partner before or at their first session, and a small chocolate as an incentive to return the completed questionnaires. Following the first session, counsellors assessed each couple’s eligibility and recommended excluding those who met the exclusion criteria stated above.

To facilitate the process, our research staff regularly visited each participating RAV site to collect forms, review recruitment processes, gather feedback on the project, and trouble-shoot any recruitment difficulties experienced by agency staff. To encourage and thank the recruiting staff, researchers often provided lunch or morning-tea for RAV staff. Between visits, regular contact was maintained with participating RAV staff by phone and e-mail.

After the first three months of recruitment, the return rate of completed questionnaires was less than anticipated. Therefore, two additional strategies were used to enhance recruitment and data collection. Firstly, express mail envelopes were used to ensure that recruitment packages reached participants before their first appointment, and to emphasise the importance of the study. Secondly, each participant was offered a $25 shopping voucher incentive, available on completion of the baseline questionnaires. These strategies improved response rates. However, due to high work pressures within the agency settings, no data were recorded by the administration staff on the number of clients who were screened out or failed to be sent information about the research. While this is a methodological limitation, the primary validity comes from our pre-post comparisons.

#### Stage 3: Retention of the sample

To increase retention rates for the three- and 12-month follow-up surveys, monetary incentives were offered in the form of department store gift-vouchers. Participants were each sent a $50 voucher for each of the two post-test questionnaires completed.

### Measures

Baseline, three and 12-month post-treatment self-report questionnaires were developed for each of the counselling and GC groups. For the baseline questionnaire there were four sets of items: socio-demographics, health characteristics, current relationship information, and reasons for attending counselling/GC course. In the three- and 12-month post-counselling questionnaires most demographic questions were omitted and additional items about their experience of the service and outcomes were included (discussed below). Slight variations existed in wording of some questions for the two groups to ensure questions were relevant to the type of service sought. For instance, ‘please consider how important each reason is for you in attending counselling’, or ‘the good connecting course’.

#### Socio-demographics

This section asked participants to provide demographic information on: birth date; gender; highest education (response options: did not complete secondary school, completed secondary school, trade or certificate, undergraduate degree, postgraduate degree); employment status (whether they did any of the following types of paid work: any paid work, shift work, night work, paid work from home, self-employment, casual work, work in more than one job); country of birth for participants and their parents (Australia, other English speaking country, Africa, Asia, Europe, Middle East, South America). Participants were asked how they manage on their income on a 5 point scale from *it is impossible* to *it is easy*.

#### Health characteristics

The health section enquired about participants’ general health, well-being and their use of health services, and included the following scales.

##### Global self-rated health

Participants rated their general health on a 5-point likert scale from *Excellent* to *Poor,* based on the global self-rated health question in the SF-36 [53].

##### Heath service use

Participants were asked how many times they have visited a medical doctor for their own health in the last 12 months, and whether they have taken medication in the past month for depression, for nerves/anxiety/worries, or to help them sleep, based on questions from the Australian Longitudinal Study on Women’s Health [54,55].

##### Life events

This section asked participants whether they have experienced any of the following 11 life events in the last 12 months (baseline), or since finishing counselling (post-tests). The events were: major illness, birth of a child, death of a family member/close friend, loss of job, retirement, major personal achievement, death of a child, miscarriage, renovation or moving house, legal troubles or involved in a court case, or other. The number of life events experienced was summed.

##### Center for Epidemiological Studies Depression scale (CESD-10)

The CESD-10 was included to assess depressive symptoms [56,57]. This 10 item scale asks participants how frequently they experienced symptoms over the past week (answered on a 4-point likert scale from *rarely/none of the time* to *most or all of the time*). The CESD-10 scores range from 0 – 30, with higher scores indicating higher levels of depression. The 10-item CESD has demonstrated sound reliability and validity (test-retest reliability of .72, internal consistence of .71) [57], and these findings have been replicated in an Australian sample [58].

#### Current relationship information

This section assessed factors related to participants’ current relationship. These items included at baseline: length of their relationship (in years); relationship status (married, living together in a relationship, separated, divorced), who else lived with them, and duration of problems in the relationship (< 6 months, < 12 months, 1–2 years, 2–5 years, > 5 years).

##### Couples satisfaction index – 32 (CSI-32)

The CSI-32 was included as a standardised assessment of relationship satisfaction [17].The CSI-32 consists of 32 self-report items, scored on a 5 point likert scale, with higher scores denoting higher relationship satisfaction [17]. The CSI-32 was created by combining the best items from previous satisfaction measures (e.g. the Marital Adjustment Test, and the DAS) [17], which supports the face validity of the scale. Further, the CSI-32 has sound predictive validity when compared to previous scales [17].

##### Commitment

To assess relationship commitment five items were adopted from the *Commitment Inventor****y*** (CI) [19]. Responses were given on 5-point scale ranging from *Strongly disagree* to *Strongly agree*, with higher scores denoting higher commitment. The included items were: ‘I want this relationship to stay strong no matter what rough times we may encounter’, ‘I want to keep the plans for my life somewhat separate from my partner’s plans for life’, ‘I get satisfaction out of doing things for my partner, even if it means I miss out on something I want for myself’, ‘my friends want to see my relationship with my partner continue’, and ‘my family really wants this relationship to work’.

##### Inventory of interpersonal problems (IIP-32)

The IIP-32 is a 32-item self-report questionnaire, with higher scores denoting greater interpersonal problems [59]. The IIP has eight sub-scales that represent distinct interpersonal problems, with coefficient alphas ranging from .68 to .84 [59]. Participants’ interpersonal problems were measured at each survey with seven of the subscales of the IIP 32: ‘Hard to be sociable’; ‘Hard to be assertive’; ‘Too aggressive; ‘Too caring’; ‘Hard to be supportive’; ‘Hard to be involved’; ‘Too dependent’.

#### Reasons for attending counselling

Finally, clients were asked to rate the importance of 10 common reasons for attending couple counselling/education that addressed: communication issues; wanting to improve the relationship, or resolve conflict; serious reasons such as to discuss future of relationship, separation, or an affair; parenting/family issues such as parenting or step parenting concerns, work/life balance, family background; and intimacy/sexuality issues. Clients rated these on a 5-point likert scale from ‘*not at all important*’ to ‘*very important*’.

#### Post-test questionnaires

In the follow-up questionnaires, the CESD-10, CSI, CI, IIP, participants’ health characteristics, and current relationship information were reassessed. We also added items on the number of counselling sessions attended, and participant’s experience in counselling. Specifically, we asked them to rate from ‘*strongly disagree*’ to ‘*strongly agree*’ factors that they felt needed more emphasis in counselling (e.g. ‘discuss gambling issues more’), any changes to their life as a result of counselling (e.g. ‘I learnt more about myself from counselling’), their feelings about their counsellor (e.g. ‘my counsellor was a likable person’), and an overall assessment of the counselling process.

### Data analysis

Firstly, preliminary analyses will be conducted to assess the data, profile and compare participants accessing counselling vs. GC services, and compare the attrition versus retained sample. Secondly, the analysis will evaluate the outcomes of couple counselling and the GC course using a Generalized Linear Latent and Mixed Modelling (GLLAMM) approach [51,60].

#### Preliminary analyses

Descriptive and comparison statistics (CC vs. GC) will be performed on all variables using the SPSS-19 statistics package. For the continuous variables independent samples *t*-tests will be used for males and females separately. For the remaining variables, *χ*^2^ tests will be applied by gender [61]. To compare baseline characteristics of the counselling and GC groups, independent samples *t*-tests and *χ*^2^ tests will be used by gender. Further, to establish whether participant attrition resulted in any meaningful differences between the attrition and test groups, between-groups comparisons will be conducted.

#### GLLAMM

**Figure 2:**
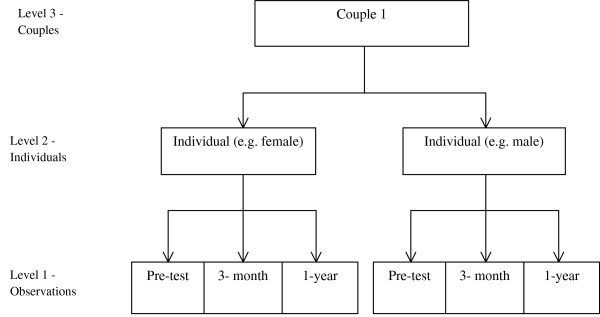
The three-level nested study design of the effectiveness of couple counselling study.

To ascertain how the identified variables (gender, age, marital status, number of counselling sessions attended, length of relationship, length of problems in relationship, reasons for attending couple counselling) influence our outcome variables (couple satisfaction, commitment, depression) at the two follow-ups, and in comparing the baseline data between groups, a generalized linear latent and mixed model (GLLAMM) is planned [51,60]. The proposed model is best understood as a more sophisticated construction of a generalized linear model (GLM), which encompasses a large number of familiar regression models. Unfortunately, basic GLMs fail when the collected data are not independently and identically distributed (i.i.d.), as in the case of all longitudinal and clustered studies, including those investigating couples. In this study, the outcomes are measured repeatedly at pre-test, 3-month and 1-year follow-up. As such, the data exhibit a hierarchical structure, i.e., the repeated observations are nested within individuals, and the individuals are nested within couples. Figure 2 illustrates the nested design of our study. GLLAMM promises to generate more reliable and precise results given the nature of the data. GLAMM will also be used in modelling baseline data to account for non-independence of the couple data. The baseline model uses 2 levels, individual and couple. All statistical tests will use Stata 11.0 (Stata Corp, Texas, U.S.A.), and adopt a 5% level of significance.

### Ethical issues

Ethical approval for the study was obtained from the La Trobe University Human Research Ethics Committee and the Relationships Australia Victoria Ethics Committee. Several key ethical issues are addressed in our methodology. Firstly, participants are fully informed about the study via the information sheet, and their written consent to participate is obtained. Secondly, as participants may be seen as in a ‘dependent’ relationship with RAV, they must be informed that participation is voluntary, they can withdraw at any time, and a decision not to participate would not affect their access to relationship services. Participant confidentiality is protected during data collection and analysis by use of participant codes. The inclusion of monetary incentives was deemed ethical, given the burden of questionnaire completion. Finally, completing the questionnaires about their relationship may raise awareness of emotional issues, and cause some participants distress. Thus, participants who need support are encouraged to contact their counsellor, or a 24 h telephone crisis line (e.g. Lifeline).

## Discussion

This ECC study is the first large scale investigation of the effectiveness of relationship counselling services to be conducted in Australia. Its significance lies in mapping client profiles, comparing the characteristics of couples who seek counselling versus relationship education, evaluating relationship service outcomes and determining the key predictors of outcomes. It builds on prior research by using a naturalistic sample, an effectiveness research design, a more robust measure of relationship satisfaction, robust health indicators, a 12-month follow-up period, and a more rigorous statistical procedure suitable for dyadic data.

The results will contribute to building an evidence base to underpin the provision of effective relationship services. In particular, the study will offer the first comparison of profiles of couples seeking different types of relationship services. Clients tend to self-select into service types when accessing assistance for relationship issues. Little is known about the client characteristics and relationship distress profiles that are the result of this self-selection process. Little is also known about the mental health and wellbeing of couples attending these services in the community. In the past, relationship education services and studies have largely targeted pre-marriage or very early relationship issues and may not address the full range of participant profiles [47,48]. Having a better understanding of participant profiles will allow for more targeted program development as well as guiding decisions about when a couple may be more likely to benefit from counselling or relationship education. It will also allow a better understanding of the relationship between relationship discord and depression and general wellbeing, and how health and community services can work together to address these co-existing health and wellbeing issues.

Another benefit of the study is its capacity to compare understandings of the relationship within couples. Such information can help to inform counsellors about areas of discrepancy and allow for analysis of the relationship between discrepancy and outcomes. Research suggests that higher within-couple discrepancy, and resulting increased discord, may be associated with poorer outcomes [8]. Little is currently known about how within couple discrepancy relates to depression and wellbeing. The use of multi-level modelling will allow for dyadic differences to be accounted for in such outcome analyses.

The most significant outcomes of the study will be the effectiveness evaluation, and a better understanding of the factors which predict improved outcomes. The study design includes a broad range of potentially relevant individual and couple relationship factors that will help to profile those couples most likely to benefit in community-based settings. Such information will assist service providers to develop more focused approaches to particular couple profiles. The study may also pave the way to develop better screening measures for couples entering relationship services, so that they can be directed to receive the most appropriate interventions.

### Real world applicability

The strengths of this investigation can also be considered limitations, due to its status as an ‘effectiveness’ study [32]. Most couple counselling research has used efficacy study designs and applied structured therapeutic approaches [62]. By not randomly assigning participants to different experimental groups, using standardised treatment protocols, or targeting participants who meet a strict profile, some will argue that the internal validity of our study is reduced [30,32]. Conversely, efficacy studies may not reflect the real world of agency-based service provision [16]. In community-based service settings, a variety of professionals will be found using different therapeutic approaches. Thus, the focus of couple counselling research is shifting from directly comparing therapies, to investigating more common factors underpinning all therapies. Research is also increasingly focused on evaluating how effective therapy can be with different types of relationship difficulties (e.g. severity, longevity, type) and for which couples (e.g. age, gender and role orientation, ethnic, geographic and economic background, life stage, recent life events). Addressing these issues is important, as effective couple counselling needs to be tailored to suit each presenting couple, and researchers are beginning to address these complex questions.

As an effectiveness study, the ECC study seeks to investigate current practice within an established service provider, and develop recommendations that may inform future training, practice, and research. Specifically, our study will take place in ‘working’ couple counselling agencies, and include a variety of clients and counselling styles, potentially enhancing the external validity of our study [30]. Therefore, the findings of effectiveness research such as this study can be viewed as a complement to previous efficacy studies.

### Collaboration to improve relationship services in Australia

The study represents a collaborative partnership with a major provider of relationship services and thus has high ecological validity. RAV is a well-established provider of community-based relationship services, and, therefore, is an ideal setting for the study of relationship services and client profiles across a variety of geographical and socio-economic regions including rural areas which have received little previous attention in relationship research. The collaboration provides the potential for results to be integrated in future policy and service delivery, and lead to a better understanding of the interaction between health and relationship indicators. This has implications for the integration of health and community services.

## Summary and conclusion

To summarise, the ECC study will investigate relationship services across Victoria, Australia. Our aims are to map profiles of clients seeking couple counselling or relationship education, determine the short- and long-term outcomes of these services, and assess the contribution of specific client factors to therapeutic outcomes. The EEC study will be an effectiveness investigation, conducted in Relationships Australia Victoria agencies, and applying statistical procedures designed for use with couple data. The study will highlight the interface between health, wellbeing and relationships, and contribute clinically applicable data to the couple counselling/relationship service literature.

## Competing interests

The fifth author is currently CEO of Relationships Australia Victoria, the setting for the study, (previously Research Director). Relationships Australia Victoria provided funding as an Australian Research Council Linkage Grant partner in the project. While he was actively involved in the design of study and facilitating access to the setting, there was no inappropriate influence on the design or conduct of the study. There are no other competing interests.

## Authors’ contributions

Prof MS had primary oversight of the project and contributed significantly to design and methodology, supervision of staff and students, and writing of study protocol. Dr NM contributed significantly to the preparation of manuscript and planning and implementation of the 12 month outcomes data collection. DJ and IJ each contributed significantly to design and survey development, gaining ethics approval, and the baseline and three month data collection. Dr AB (CEO of Relationships Australia Victoria) contributed to study design and manuscript preparation, facilitated access to relationships services and recruitment processes, and supervised staff and students. All authors read and approved the final manuscript.

## Pre-publication history

The pre-publication history for this paper can be accessed here:

http://www.biomedcentral.com/1471-2458/12/735/prepub
